# The Treatment of Inoperable Cancer of the Cervix

**Published:** 1910-03-26

**Authors:** 


### SPECIAL ARTICLES.

### THE TREATMENT OF INOPERABLE CANCER OF THE CERVIX.

Cancer of the cervix uteri is still the most hopeless of the common forms of cancer, partly because in the early stages the symptoms are not sufficiently striking to induce the patient to seek treatment, and partly because of the anatomical difficulties in the way of completely removing the whole growth with its lymphatic connections, even in "favourable cases."

The last state of a patient affected with uterine cancer is unhappy in the extreme—tortured by pain, wearied with sleeplessness, exhausted by hæmorrhage and discharge, death is a welcome release from intolerable suffering; and the end does not come pleasantly as in many diseases. A terrifying death from hæmorrhage, a terminal peritonitis, or a slow toxæmia is an unhappy termination which robs death of none of its terrors.

The chief symptoms are pain, hæmorrhage, and discharge, but vomiting is often a troublesome feature—curious, causeless vomiting, unassociated with the question of food, and similar in many ways to the vomiting of pregnancy.

The bleeding may be in the nature of a constant oozing, which gradually blanches the patient; or there may be an occasional sharp burst of hæmorrhage due to the opening of some considerable vessel, possibly the uterine artery itself. Discharge is copious, and always offensive, for infection of the growth is inevitable; drainage is imperfect, and sloughing of portions of the neoplasm is certain to occur. In these dead and dying tissues saphrophytic organisms flourish abundantly, and toxæmia follows as a matter of course.

So constant and so active are these septic processes that it is quite exceptional to meet with a case in which there is massive growth. In the vast majority the neoplasm ulcerates and sloughs away almost *pari passu* with its formation—indeed, it is no uncommon thing to find that in a patient dead of a malignant growth of the cervix the actual visible cancer is no larger than a five-shilling piece. It is to the prevention or limitation of septic changes that one's efforts should chiefly be directed in attempting to render these patients comfortable.

A detailed post-mortem examination of a typical case shows the upper portion of the vagina and the lower half of the uterus to be replaced by a foul ulcerating chasm, probably extending into the bladder in front and perhaps invading the rectum posteriorly.

The fundal portion of the uterus usually escapes, and is found—a rounded knob of relatively normal tissue—bound down in the pelvis by adhesions to the contracted and infiltrated broad ligaments. Occupying the pouch of Douglas, there may be an abscess cavity, shut off from the general peritoneum by adherent coils of small intestine. On the posterior wall of the abdomen the ureters will be seen—tortuous, dilated tubes distended with urine dammed back by obstruction in the pelvis. It is instructive that the left ureter usually shows the more marked degree of this change, possibly owing to the greater amount of cellular tissue on the left side of the pelvis favouring extension of infiltration from the uterus. On the side walls of the pelvis along the iliac vessels enlarged lymphatic glands will be found, firmly adherent to the surrounding structures. The most interesting part of the examination is to come, however, for when microscopical investigation of the enlarged glands, of the infiltrated broad ligaments, and of the cellular tissues is carried out, it is found that their histological features are not those of new growth, as might be expected, but are those of inflammation.

And here we have the whole pathological history of cancer of the cervix. It is not the cancer that kills, it is the sepsis and the septic absorption which accompany its growth that are so lethal.

The uterus is not fixed and bound down by carcinomatous permeation, but by inflammatory infiltration; the ureters are not blocked by the contraction of the connective-tissue stroma of the growth, but by inflammatory exudate in their walls—a fact which no doubt explains the frequency with which ascending pyelo-nephritis complicates these cases.

The control of this sepsis is the only thing that prolongs the life of patients with cervical cancer. They may be kept relatively clean by constant douching with antiseptic lotions; but this is not enough. Three things are open to us: Either the growth may be starved and its progress limited by shutting off its blood supply. This entails opening the abdomen and tying the uterine arteries—a formidable undertaking in a patient teeming with infective organisms and in the worst possible condition to stand an abdominal operation of considerable difficulty; for it is not easy to find, let alone to ligature, the uterine arteries under these circumstances.

The sloughing masses of growth may be removed by curetting. The patient is anæsthetised, placed in the lithotomy position, and thoroughly examined. If there is a large mass of growth it is cut away—an extremely easy matter if, as sometimes happens, it springs, cauliflower-like, from a narrow base. If, on the other hand, as is more usual, there is an irregular friable growth of small size, it is curetted away with a sharp spoon. Hæmorrhage is free, but it is controlled by means of douches of hot water (115°-120° F.), and by the pressure of dabs held in long forceps. This will bring about considerable amelioration in the symptoms, but unfortunately many organisms are still there, growth will continue, and the patient will soon be as bad as ever. A distinct advance has quite recently been made by the introduction of the use of acetone in these cases. Acetone is a very volatile liquid having properties very similar to absolute alcohol, but it is much more hygroscopic and much more penetrating, consequently it is greatly used in histological work for hardening tissues. It is rational, therefore, to suppose that, applied to a new growth *in situ*, it will to a certain extent exercise these well-marked hardening powers. It is, at the same time, a powerful germicide, so that not only does it destroy organisms, but it renders the tissues in which they were present unsuitable for their development.

Its application is a simple matter. After the main mass of growth has been curetted away and the hæmorrhage has ceased, a Ferguson's speculum is inserted and a quantity of pure acetone is poured on to the growth, where it is allowed to remain ten minutes, by which time the surface of the ulcer will be thoroughly blanched and hardened, just as if it were being prepared for microscopical examination. Care should be taken that excess of acetone is removed, as it causes considerable irritation if it comes in contact with the healthy portion of the vagina.

A case of advanced cancer of the cervix treated by this method is given a greater degree of relief from the more obnoxious symptoms of the disease than by any other means at present at our disposal.

The ideal method of treatment of these cases—the administration of the mixed vaccines of the numerous organisms isolated from the vagina has not yet, it would appear, been attempted, and perhaps is not at present within the realms of practical bacteriology. When the time does come for this to be feasible a very great advance will have been made in the treatment of inoperable cancer.

